# Neutrophil extracellular traps and pyroptosis: a molecular nexus linking systemic autoimmune diseases to atherosclerosis

**DOI:** 10.3389/fimmu.2026.1796827

**Published:** 2026-05-12

**Authors:** Yan Du, Lulu Zhang, Yiheng Cheng, Sijie Lu, Ronghua Ou, Xiaojian Deng

**Affiliations:** 1 Department of Cardiology, People's Hospital of Deyang City, Deyang, Sichuan, China; 2 Department of Paediatrics, People's Hospital of Deyang City, Deyang, Sichuan, China

**Keywords:** atherosclerosis, autoimmune diseases, extracellular, gasdermins, neutrophil, pyroptosis, systemic

## Abstract

This review aims to elucidate the molecular mechanisms of neutrophil extracellular traps (NETs) and pyroptosis, exploring their synergistic role as a pathological nexus bridging systemic autoimmune diseases with atherosclerosis. By systematically synthesizing recent literature and experimental evidence, we delineate the signaling cascades of these programmed inflammatory processes and analyze their contributions to disease progression in systemic lupus erythematosus, rheumatoid arthritis, and cardiovascular pathology. The analysis reveals a sophisticated bidirectional crosstalk, termed the NET-pyroptosis axis, which functions as a self-amplifying inflammatory loop that drives chronic tissue injury. Key findings highlight that while Gasdermin D serves as a context-dependent amplifier of NET release rather than a universal executioner, the Gasdermin E-mediated interaction between immune and stromal cells emerges as a critical driver of structural damage, such as synovial bone erosion and endothelial plaque denudation. Furthermore, we summarize emerging therapeutic strategies, including the pharmacological inhibition of peptidylarginine deiminase 4 and gasdermin pore formation, which hold significant promise for mitigating both systemic inflammation and the associated cardiovascular burden. Ultimately, this review establishes a novel pathophysiological framework that explains the heightened cardiovascular risk in autoimmune populations and provides a rationale for cross-disciplinary clinical interventions. Targeting the reciprocal interaction between these two inflammatory pathways may offer transformative breakthroughs in improving the long-term prognosis of patients with chronic inflammatory disorders.

## 1 Introduction

Neutrophils represent the most prevalent class of leukocytes in human circulation. Serving as the primary line of defense within the innate immune system, these cells play a pivotal role in host protection against pathogenic invasion (1). Conventionally, neutrophil-mediated antimicrobial activities were considered to be restricted to phagocytosis, degranulation, and the generation of reactive oxygen species (ROS). However, a landmark study in 2004 unveiled a previously unrecognized defense mechanism: the formation of neutrophil extracellular traps (NETs) (2).

NETs are extracellular web-like structures composed of a decondensed chromatin DNA backbone. These lattices are decorated with an array of granular and cytoplasmic proteins, such as myeloperoxidase (MPO), neutrophil elastase (NE), histones, and antimicrobial peptides (3, 4). This specialized architecture facilitates the efficient entrapment and subsequent neutralization of a broad spectrum of pathogens, including bacteria, fungi, viruses, and parasites, thereby serving as a vital component of the host’s immune repertoire.

The process of NET formation, known as NETosis, represents a specialized form of regulated cell death that is mechanistically distinct from apoptosis and necrosis (5). While indispensable for host defense, this “double-edged sword” of innate immunity has increasingly been implicated in the deleterious progression of various non-infectious pathologies (6, 7). Mounting evidence suggests that the aberrant accumulation of NETs—stemming from either exaggerated production or defective clearance—is fundamentally linked to the pathogenesis of autoimmune disorders, thrombosis, organ injury, and malignancy (8–10).

In the context of systemic autoimmune diseases, such as systemic lupus erythematosus (SLE) and rheumatoid arthritis (RA), NETs function as a prominent reservoir of autoantigens, thereby perpetuating and amplifying the autoimmune response (11). Furthermore, NETs exacerbate cardiovascular complications, most notably atherosclerosis, by compromising endothelial integrity and fostering a pro-thrombotic environment. Consequently, NETs emerge as a critical mechanistic nexus bridging systemic autoimmune inflammation with cardiovascular pathology (11).

Concurrently, advances in cell death research have identified pyroptosis as another distinct form of programmed inflammatory cell death. This process is primarily driven by inflammasome activation, which triggers the proteolytic cleavage of the gasdermin protein family, most notably Gasdermin D (GSDMD). These molecular events culminate in the formation of transmembrane pores and the rapid secretion of pro-inflammatory cytokines, specifically IL-1β and IL-18, thereby orchestrating a robust inflammatory response (5).

Accumulating evidence points toward a sophisticated interplay and regulatory crosstalk between NETosis and pyroptosis. Collectively, these pathways constitute a self-amplifying inflammatory loop that serves as a central driver in various pathophysiological settings (12, 13). Consequently, elucidating the molecular mechanisms of NET formation, its synergistic interaction with pyroptosis, and their combined contribution to systemic autoimmune diseases and atherosclerosis is imperative for the development of targeted therapeutic strategies.

This review provides a systematic delineation of the molecular architecture and biological hallmarks of NETs, while exploring their role as a critical pathological nexus between systemic autoimmunity and atherosclerosis. Furthermore, we summarize emerging therapeutic strategies and translational advancements targeting NETs and their constituent signaling pathways to highlight potential clinical interventions.

## 2 Molecular mechanisms of NET formation

NETosis is a tightly regulated process categorized into two distinct modalities based on the eventual fate of the neutrophil: suicidal and vital NETosis (8).

### 2.1 Classical (suicidal) NETosis

Suicidal NETosis, the canonical form of this defense mechanism, typically transpires over several hours and culminates in the programmed death of the neutrophil. This pathway is triggered by diverse stimuli, including pathogen-associated molecular patterns (PAMPs), damage-associated molecular patterns (DAMPs), immune complexes, phorbol 12-myristate 13-acetate (PMA), and autoantibodies (14).

The signaling cascade is initiated upon the engagement of Toll-like receptors (TLRs) or Fcγ receptors, which facilitates the assembly and subsequent activation of the NADPH oxidase (NOX2) complex. The ensuing generation of ROS serves as a pivotal requirement for downstream signaling (15). Following this oxidative burst, ROS mediate the translocation of the calcium-dependent enzyme peptidylarginine deiminase 4 (PAD4) from the cytoplasm to the nucleus. PAD4 catalyzes the deamination of arginine residues on histones (primarily H3 and H4) into citrulline. This histone citrullination neutralizes the electrostatic attraction between histones and DNA, triggering the unwinding of highly condensed heterochromatin (16).

Concurrently, NE and MPO translocate from azurophilic granules to the nucleus. NE promotes chromatin decondensation by partially degrading histones, a process synergistically enhanced by MPO (14). The eventual disintegration of both the nuclear and plasma membranes facilitates the liberation of decondensed chromatin and sequestered granular proteins into the extracellular space. The resulting NETs comprise a DNA scaffold decorated with various effector molecules, including MPO, NE, the antimicrobial peptide LL-37, and high mobility group box 1 (HMGB1). Beyond pathogen entrapment, these structures serve as potent pro-inflammatory platforms that activate adjacent immune cells and exacerbate the systemic inflammatory response.

### 2.2 Vital NETosis

Distinct from suicidal NETosis, vital NETosis is a rapid, non-lytic process that transpires within minutes while preserving neutrophil viability. Subsequent to NET release, these neutrophils maintain their plasma membrane integrity and retain essential physiological functions, such as chemotaxis and phagocytosis (17). The induction of vital NETosis is typically triggered by the activation of the complement system or through direct interaction with platelets. Based on the origin of the extracellular genomic material, vital NETs are categorized into two distinct types: those derived from nuclear DNA (nDNA)—wherein nuclear components are expelled via vesicular trafficking—and those originating from mitochondrial DNA (mtDNA).

Under specific stimulatory conditions, such as the synergy between granulocyte-macrophage colony-stimulating factor (GM-CSF) and complement C5a, activated neutrophils selectively extrude their mitochondria. These organelles subsequently undergo disintegration in the extracellular space, liberating mtDNA to form mitochondrial NETs (mtNETs) (17). Due to its evolutionary prokaryotic signatures, such as unmethylated CpG motifs, mtDNA exhibits heightened immunogenicity and potently activates pattern recognition receptors, notably Toll-like receptor 9 (TLR9). In clinical contexts such as SLE, NETs enriched with oxidized mtDNA have been demonstrated to robustly induce type I interferon production, thereby acting as primary drivers of autoimmune inflammation (18).

### 2.3 Re-evaluating the role of GSDMD in NETosis: from “obligate executioner” to “context-dependent amplifier”

In initial molecular frameworks, GSDMD was characterized as the universal executioner protein bridging neutrophil pyroptosis with the terminal phases of NETosis. A seminal 2018 study postulated that the proteolytic cleavage of GSDMD by NE—yielding pore-forming N-terminal fragments—served as the definitive physical prerequisite for chromatin extrusion (19). However, a wave of independent studies published between 2022 and 2024 has leveraged rigorous genetic approaches to challenge this notion of “absolute necessity,” revealing that NETosis execution is far more complex and stimulus-dependent than previously recognized.

Specifically, Chauhan et al. (2022) demonstrated, using GSDMD-deficient murine models, that while GSDMD remains indispensable for classical inflammasome-driven neutrophil pyroptosis (characterized by rapid cytolysis and IL-1β secretion), it is not strictly required for chromatin decondensation or extracellular release—the defining hallmarks of NETosis (20). Their findings revealed that GSDMD-deficient neutrophils can still form morphologically intact NETs upon stimulation with PMA or immune complexes, indicating that chromatin extrusion can proceed independently of GSDMD-mediated membrane perforation. Furthermore, Stojkov et al. (2023) confirmed that under specific stimuli, such as PMA or bacterial pore-forming toxins, NET formation is entirely decoupled from GSDMD activation and the pyroptotic cascade (21). Real-time imaging suggests that in the absence of GSDMD-mediated pores, neutrophils may leverage the physical pressure generated by explosive chromatin expansion to mechanically rupture cellular membranes, or alternatively, employ hitherto unidentified pore-forming mechanisms to facilitate DNA expulsion.

In light of these contemporary findings, this review advocates for a revision of the conventional linear pathway, repositioning GSDMD as a context-dependent inflammatory modulator. While GSDMD-mediated perforation remains the primary driver for rapid NET release in non-canonical inflammasome pathways, its role in autoimmune-driven NETosis—such as in SLE or RA—appears to be that of an “inflammatory intensity amplifier” and a gatekeeper for cytokine secretion, rather than a universal prerequisite for DNA ejection. This paradigm shift provides a robust theoretical framework for precision therapeutic strategies, necessitating a clinical distinction between targeting the GSDMD axis and the upstream ROS-PAD4 pathway based on the specific inflammatory context.

## 3 Molecular interplay between pyroptosis and NETosis

Pyroptosis is a programmed modality of inflammatory cell death defined by its reliance on inflammasome activation and the gasdermin protein family [reviewed in 5]. Upon recognition of pathogenic or endogenous danger signals, cells orchestrate the assembly of inflammasome complexes (e.g., NLRP3 or AIM2), which subsequently activate inflammatory caspases, primarily caspase-1. These activated caspases mediate the proteolytic processing of GSDMD, liberating its bioactive N-terminal fragment (GSDMD-N). This fragment oligomerizes on the plasma membrane to construct transmembrane pores (10–20 nm in diameter), precipitating osmotic dysregulation, cellular tumescence, and eventual cytolysis. This cascade facilitates the release of mature IL-1β, IL-18, and other intracellular contents, orchestrating a robust inflammatory response (22).

While historically viewed as discrete cell death pathways, emerging evidence underscores a sophisticated interplay and reciprocal positive feedback between NETosis and pyroptosis. Beyond its canonical role as the executioner of pyroptosis, GSDMD serves as an integral mediator of NET formation. Evidence indicates that GSDMD-N targets not only the plasma membrane but also endomembranes, including granular and nuclear envelopes. These pores permit the nuclear translocation of granular proteases, such as NE, which facilitates chromatin decondensation and accelerates the kinetics of NET release (23). Intriguingly, NE can reciprocally process GSDMD to further potentiate its pore-forming capacity, establishing a feed-forward loop that substantially amplifies both NET production and the ensuing inflammatory cascade (13). Furthermore, within the non-canonical inflammasome pathway, intracellular lipopolysaccharide (LPS) triggers caspases 4, 5, or 11 to activate GSDMD. This process similarly accelerates NET release and promotes the exposure of pro-coagulant determinants (24). Consequently, GSDMD has emerged as a pivotal molecular nexus bridging the inflammatory programs of pyroptosis and NETosis.

However, recent pioneering studies have contested this linear paradigm by questioning the “obligate requirement” for GSDMD in NET formation. Recent independent studies utilizing rigorous GSDMD-deficient murine models have demonstrated that neutrophils can still form morphologically intact NETs upon stimulation with PMA or specific immune complexes (20, 21). These findings highlight the “context-dependent” plasticity of the NETosis execution machinery (20). While GSDMD remains the primary driver of rapid NET release in non-canonical pathways triggered by intracellular bacteria (25), in autoimmune-driven classical NETosis, the substantial mechanical pressure generated by ROS-mediated chromatin expansion may drive DNA expulsion independently of GSDMD. This process may be facilitated by alternative pore-forming proteins, such as MLKL, or driven by mechanical membrane rupture. Thus, GSDMD should be redefined as an amplifier of “lytic inflammation” rather than a universal prerequisite for all NET formation (26).

Notably, the repertoire of pyroptotic executioners extends beyond GSDMD; specifically, gasdermin E (GSDME) serves as a critical conduit between immune-mediated inflammation and structural tissue damage (27). This “immune–stromal interaction” elucidates why NETs contribute to direct tissue degradation rather than acting solely as signaling amplifiers (28). In the joint microenvironment of RA, NETs activate the NF-κB signaling pathway within fibroblast-like synoviocytes (FLSs), inducing caspase-3-mediated GSDME cleavage. This transition triggers a phenotypic shift in cell death from immunologically silent apoptosis to pro-inflammatory pyroptosis (29). The ensuing liberation of DAMPs, such as HMGB1 and ATP, from pyroptotic FLSs robustly stimulates osteoclastogenesis, directly contributing to bone erosion—a paradigm-shifting perspective on joint destruction in RA.

Similarly, in atherosclerosis, circulating NETs act synergistically with oxidized low-density lipoprotein (oxLDL) to activate the caspase-3/GSDME axis through mitochondrial dysfunction in endothelial cells. This culminates in the compromise of endothelial barrier integrity and subsequent plaque erosion (30, 31). This mechanism delineates plaque erosion from rupture, highlighting the decisive role of NETs in endothelial denudation. This mechanistic distinction is fundamental to understanding the distinct clinical phenotypes observed in younger or female AS patient cohorts (32). Additionally, the role of NETs as physical scaffolds in “immunothrombosis”—capturing platelets and activating coagulation factors—elucidates the refractoriness of autoimmune-associated thrombosis to conventional anticoagulants (33).

## 4 NETs in systemic autoimmune diseases

Systemic autoimmune diseases comprise a spectrum of chronic inflammatory disorders characterized by a loss of self-tolerance and aberrant immune responses against host tissues and organs. In these pathologies, NETs exert multifaceted effects, functioning as a primary reservoir of autoantigens, potent amplifiers of the inflammatory cascade, and direct executioners of structural tissue damage [reviewed in 34].

### 4.1 SLE: nucleic acid sensing, interferon signatures, and renal sequelae

The pathogenesis of SLE is fundamentally driven by the breakdown of immunological tolerance toward endogenous nucleic acids. Neutrophils from SLE patients exhibit profound functional aberrations and are highly prone to exaggerated NETosis, a process primarily triggered by low-density granulocytes (LDGs) and autoantibodies, such as anti-RNP (35). Compounded by impaired serum DNase I activity, these persistent NETs serve as a sustained reservoir for autoantigens, notably double-stranded DNA (dsDNA) and nucleosomes (36–38).

Within the pro-inflammatory microenvironment of SLE, defective ROS scavenging facilitates the liberation of NETs enriched with oxidized mitochondrial DNA (Ox-mtDNA) (18). Compared to genomic DNA, Ox-mtDNA possesses superior immunogenicity. Complexes composed of Ox-mtDNA and the antimicrobial peptide LL-37 not only activate TLR9 in plasmacytoid dendritic cells (pDCs) but also facilitate cytosolic translocation to trigger the cGAS-STING pathway (18). This signaling cascade culminates in a robust release of type I interferon (IFN-α), characterizing the “interferon signature” observed in SLE. This interferon surge, in turn, sensitizes neutrophils to further NETosis, establishing a self-perpetuating pathogenic loop (39, 40).

The synergistic interplay between NETs and pyroptosis serves as a primary driver of organ-specific injury, most notably in lupus nephritis (LN). LL-37/DNA complexes embedded within NETs activate the NLRP3 inflammasome in macrophages, thereby prompting the secretion of mature IL-1β and IL-18 (41). Recent single-cell RNA sequencing (scRNA-seq) analysis of renal biopsies reveals that GSDMD activation and pyroptotic signatures correlate positively with proteinuria severity and declining renal function, identifying pyroptosis as a central driver of parenchymal injury. Furthermore, in SLE-associated pregnancy complications, elevated NET infiltration promotes NLRP3 lactylation, which triggers trophoblast pyroptosis and leads to adverse gestational outcomes (12).

Regarding vascular sequelae, NETs exert direct cytotoxicity on endothelial cells and precipitate endothelial dysfunction through the activation of matrix metalloproteinases, specifically MMP-2. This mechanism is central to the heightened risk of premature AS in SLE patients (41, 42). Currently, circulating NET markers—notably MPO-DNA and citrullinated histone H3 (Cit-H3)—have been validated as reliable indicators of disease activity and renal involvement, underscoring their potential as clinical biomarkers for disease monitoring and the prediction of thrombotic events (43–45).

### 4.2 RA: citrullination, synovial pyroptosis, and articular destruction

RA is a systemic autoimmune disorder characterized by symmetrical polyarthritis, chronic synovitis, and progressive osteoarticular erosion. The marked infiltration of neutrophils into the RA synovial compartment and the subsequent release of NETs are pivotal drivers of synovial inflammation and joint destruction. Within the RA-associated pathological microenvironment, neutrophil peptidylarginine deiminase 4 (PAD4) activity is pathologically upregulated. During NETosis, PAD4 not only facilitates the extensive citrullination of nuclear histones (primarily H3 and H4) but is also externalized onto the DNA scaffold into the extracellular milieu. Within the extracellular space, PAD4 post-translationally modifies various stromal proteins, notably fibrinogen and vimentin, through citrullination (46–48).

These citrullinated proteins function as neoantigens, triggering the production of high-affinity anti-citrullinated protein antibodies (ACPAs). The resulting immune complexes, composed of ACPAs and citrullinated antigens, can further prime and activate neutrophils via Fcγ receptors, thereby triggering recurrent cycles of NETosis and establishing a self-perpetuating inflammatory loop (46).

Beyond their role in antigen presentation, the pathogenic impact of NETs on the joint is further exemplified by “immune–stromal crosstalk,” which induces a phenotypic shift in synovial resident cells. Recent evidence indicates that synovial GSDME expression levels in RA patients correlate positively with systemic NET markers (29). NETs activate FLSs via the TLR4/NF-κB axis, triggering caspase-3-mediated GSDME cleavage. This molecular switch prompts a modal shift in FLS death from immunologically silent apoptosis to pro-inflammatory pyroptosis (29, 49).

**Figure 1:**
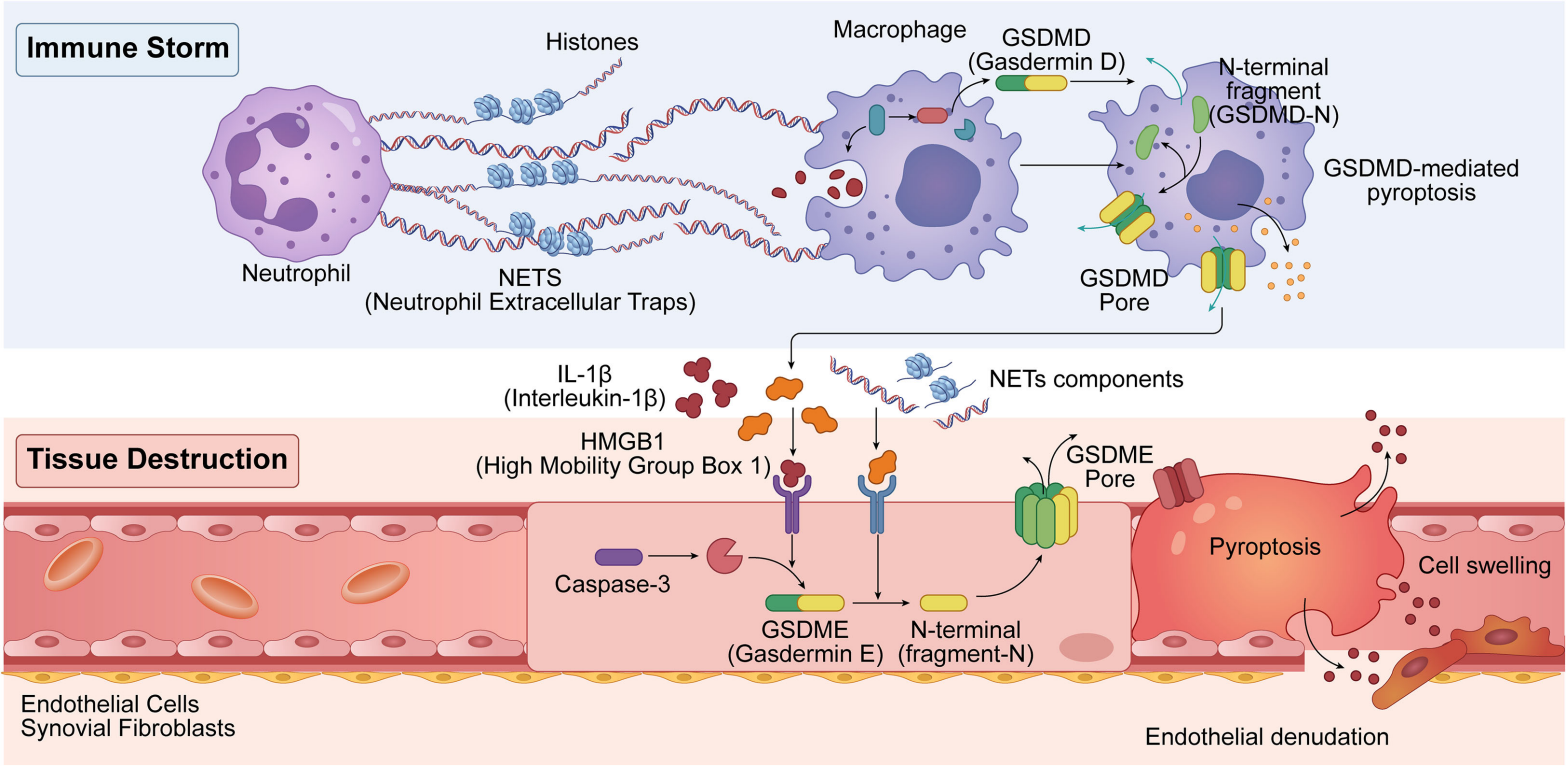
The “death switch” of tissue injury: the GSDMD–GSDME signaling relay. This schematic illustrates the molecular mechanisms of the pyroptotic cascade. The process is initiated by an “immune storm” and mediated by Gasdermin D (GSDMD) and Gasdermin E (GSDME), ultimately culminating in systemic tissue destruction. The Immune Storm (Upper Panel) The cascade begins when activated neutrophils release neutrophil extracellular traps (NETs), which are web-like structures composed of DNA fibers and proteins such as histones. These NETs serve as primary danger signals that activate macrophages. Upon activation, intracellular full-length GSDMD is cleaved to produce cytotoxic N-terminal fragments (GSDMD-N). These fragments oligomerize on the plasma membrane to form GSDMD pores, which drive GSDMD-mediated pyroptosis. Mediator Release (Middle Panel) The death of these macrophages results in the massive liberation of intracellular pro-inflammatory mediators into the microenvironment. Key factors released include interleukin-1β (IL-1β), high-mobility group box 1 (HMGB1), and residual NET components. Tissue Destruction (Lower Panel) These liberated alarmins subsequently target downstream structural cells, including endothelial cells and synovial fibroblasts. The binding of these ligands to their respective surface receptors triggers intracellular signaling pathways that activate caspase-3. Once activated, caspase-3 cleaves full-length GSDME, generating pore-forming N-terminal fragments (GSDME-N). Similar to the mechanism of GSDMD, GSDME-N forms transmembrane pores in structural cells, inducing cellular swelling and the execution of pyroptosis. This inflammatory cell death disrupts the integrity of the cellular monolayer, leading to endothelial denudation and irreversible tissue injury.

This phenotypic transition carries significant pathological implications for disease progression. Pyroptotic FLSs release not only a cascade of pro-inflammatory cytokines (e.g., IL-1β and IL-6) but also potent “alarmins” or DAMPs, including HMGB1 and ATP. These molecules serve as powerful activators of osteoclastogenesis, directly driving cartilage degradation and articular bone erosion (29, 50). This NET-initiated “FLS pyroptosis–bone erosion” cascade elucidates why local bone destruction may persist in certain clinical contexts—driven by altered cell death modalities—even when systemic inflammation is partially mitigated. These insights offer a novel therapeutic paradigm targeting the GSDME axis and its downstream signaling for precision interventions in RA (**Figure 1**).

### 4.3 Other autoimmune diseases

Beyond SLE and RA, the pathological significance of NETs extends to other autoimmune conditions, most notably antiphospholipid syndrome (APS) and ANCA-associated vasculitis (AAV). APS is characterized by recurrent venous or arterial thromboembolism and obstetric complications, pathologies fundamentally driven by the persistence of antiphospholipid antibodies (aPLs). In the context of APS, the reciprocal potentiation between pyroptosis and NETosis is particularly pronounced. aPLs trigger the activation of the NLRP3 inflammasome in monocytes and endothelial cells, mediating GSDMD cleavage through caspases 1, 4, and 5. This cascade culminates in pyroptotic cell death and the liberation of pro-coagulant determinants, such as tissue factor (TF) (51). Concurrently, aPLs directly prime and induce NETosis in neutrophils (52). These NETs provide a structural template for thrombus assembly and, in turn, promote pyroptosis in neighboring monocytes and macrophages. This crosstalk facilitates the sustained release of pro-thrombotic factors, establishing a “NET–pyroptosis–thrombosis” feed-forward loop (53, 54).

In AAV, anti-neutrophil cytoplasmic antibodies (ANCAs) recognize and bind to cognate antigens on the neutrophil surface, specifically MPO or proteinase 3 (PR3). This interaction directly triggers neutrophil activation, leading to robust ROS generation and subsequent NET release. These NETs inflict direct injury upon the vascular endothelium and orchestrate the activation of the complement system. Furthermore, MPO and PR3 anchored within the NET framework serve as persistent autoantigens, driving continuous ANCA production and perpetuating a state of chronic systemic vasculitis (55, 56).

## 5 Mechanisms of NET-mediated atherogenesis and thrombosis

AS is a chronic inflammatory pathology characterized by subendothelial lipid deposition, persistent vascular inflammation, and fibrous tissue proliferation. It serves as the fundamental pathological substrate underlying various cardiovascular diseases. Patients with systemic autoimmune disorders exhibit a significantly heightened risk of AS, with NETs serving as a critical pathological nexus bridging these two distinct disease spectrums [reviewed in 57].

### 5.1 Lipid metabolism and inflammasome activation

The atherosclerotic plaque microenvironment is characterized by a plethora of danger signals, notably cholesterol crystals, oxLDL, and hyperglycemic stimuli. These stimuli potently activate plaque-infiltrating neutrophils and macrophages, triggering the assembly and activation of the NLRP3 inflammasome (58). This activation culminates in GSDMD-mediated pyroptosis while concurrently facilitating the expulsion of NETs.

Upon liberation, NET components—specifically the DNA scaffold—are recognized by the absent in melanoma 2 (AIM2) sensor within macrophages (59, 60). This recognition triggers the activation of the AIM2 inflammasome, further driving macrophage-specific pyroptosis (13). Pyroptotic macrophages liberate significant quantities of pro-inflammatory cytokines, such as IL-1β and IL-18, which subsequently recruit additional neutrophils and sensitize them to further NETosis (61, 62). This “inflammasome-NET” feed-forward loop represents a central mechanism driving the sustained amplification of intra-plaque inflammation, lesion progression, and eventual plaque destabilization (13, 63).

### 5.2 Endothelial injury and pro-thrombotic mechanisms: plaque erosion vs. rupture

Atherosclerotic thrombosis, a major complication of advanced plaques, is fundamentally driven by the interplay between NETs and pyroptosis, which dictates the thrombotic phenotype—whether through superficial erosion or plaque rupture.

**Figure 2:**
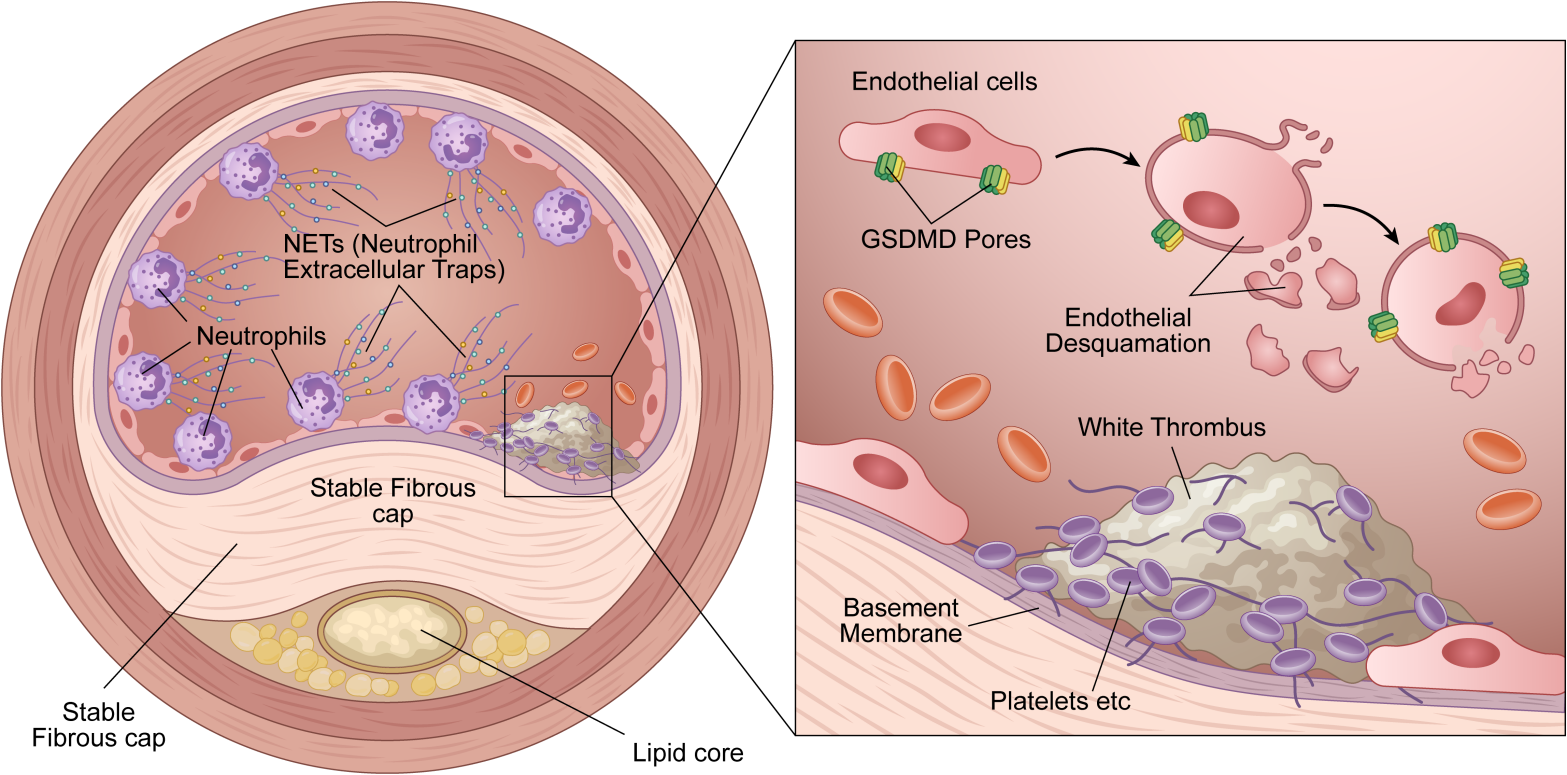
Plaque erosion: GSDME-mediated endothelial denudation. This schematic illustrates the specific mechanisms underlying superficial plaque erosion in atherosclerosis. It highlights the critical role of GSDME-mediated endothelial pyroptosis in the initiation of thrombus formation. Arterial Cross-section (Left Panel) The diagram depicts an atherosclerotic plaque characterized by a stable fibrous cap and a minimal lipid core. Within the vascular lumen, activated neutrophils aggregate near the endothelial layer and release web-like neutrophil extracellular traps (NETs), which coat the endothelial surface. Magnified View (Right Panel) This detailed view shows the pathological events at the luminal surface. NETs released by neutrophils interact with endothelial cells, triggering the formation of GSDME pores on the plasma membrane. This process induces pyroptosis, characterized by cellular swelling and rupture. Consequently, the endothelial cells detach from the underlying structure, a process termed endothelial denudation (or desquamation). Thrombus Formation The loss of endothelial integrity exposes the underlying pro-thrombotic basement membrane. Circulating platelets rapidly adhere to and aggregate at the site of exposure. This sequence culminates in the formation of a platelet-rich white thrombus, ultimately leading to vascular occlusion.

In the context of superficial plaque erosion, NETs exert direct cytotoxic effects on the vascular endothelium. Extracellular histones within the NET architecture—notably H3 and H4—possess potent cytotoxicity, compromising endothelial membrane integrity and precipitating cellular injury or apoptosis (41). Furthermore, NET-associated MPO depletes the bioavailability of endothelium-derived nitric oxide (NO), thereby impairing vasoregulatory capacity. Crucially, recent evidence highlights that histone-induced severe oxidative stress strongly activates caspase-3 in endothelial cells, which subsequently cleaves Gasdermin E (GSDME). This shifts the endothelial cell fate from immunologically silent apoptosis to highly pro-inflammatory pyroptosis, resulting in massive cell lysis, degradation of tight junctions (e.g., VE-cadherin), and widespread endothelial denudation (31, 64). This denudation directly exposes basement membrane collagen and von Willebrand factor (vWF), orchestrating a platelet- and NET-rich “white thrombus” (65, 66) (**Figure 2**).

Conversely, during classic plaque rupture, the thrombogenic cascade is primarily initiated by the NLRP3/Gasdermin D (GSDMD)-mediated pyroptosis of lipid-laden macrophages within the necrotic core (67). This pyroptotic execution involves a massive influx of calcium, leading to the externalization of phosphatidylserine (PS) and the subsequent “decryption” of tissue factor (TF) into a highly pro-coagulant conformation. The explosive release of these TF-enriched microvesicles rapidly initiates the extrinsic coagulation cascade (68). The resulting intense inflammatory gradient recruits neutrophils to the rupture site, triggering secondary NETosis to consolidate the thrombus (69–71). The distinct molecular and pathological mechanisms distinguishing the NET-pyroptosis axis in plaque rupture versus superficial erosion are comprehensively summarized in **Table 1**.

**Table 1:** Distinct molecular and pathological mechanisms of the NET-pyroptosis axis in atherosclerotic plaque rupture versus plaque erosion.

Comparative dimension	Plaque rupture	Plaque erosion
Pathological Substrate	Large lipid necrotic core, thin and vulnerable fibrous cap	Rich extracellular matrix, minimal lipid, thick and intact fibrous cap
Core Driving Immune Cells	Macrophages (foam cells) predominant	Neutrophils predominant
Dominant Pyroptotic Pathway	Macrophage pyroptosis (NLRP3/Caspase-1/GSDMD axis)	Endothelial cell pyroptosis (Mitochondrial dysfunction/Caspase-3/GSDME axis)
Primary Thrombogenic Trigger	Decryption of Tissue Factor (TF) and release of pro-coagulant microvesicles	Endothelial denudation, direct exposure of basement membrane collagen and vWF
Dominant Coagulation Pathway	High activation of extrinsic coagulation pathway (TF-FVIIa dependent)	Activation of intrinsic contact pathway interacting with strong platelet aggregation
Characteristic Thrombus Morphology	Fibrin- and erythrocyte-rich “red thrombus” (often causing complete occlusion)	Platelet- and NET-scaffold-rich “white thrombus” (often causing partial occlusion)

Beyond inflicting direct vascular injury, NETs function as highly thrombogenic platforms driving a systemic “immunothrombosis” amplification loop. Their negatively charged DNA scaffolds provide a physical substrate for platelet adhesion and coagulation factor assembly (33), and facilitate the autoactivation of Factor XII, launching the intrinsic coagulation pathway. While NET-associated histones directly activate platelets via TLR2 and TLR4, triggering platelet aggregation and granule release (72, 73), activated platelets reciprocally release high-mobility group box 1 (HMGB1), which binds to neutrophil receptors to hyperactivate further NETosis. NETs also recruit and interact with von Willebrand factor (vWF) and fibrinogen to provide a structural framework that sequesters erythrocytes, platelets, and fibrin, essentially orchestrating the core architecture of a thrombus. Concurrently, enzymes such as MPO oxidatively modify circulating lipoproteins; specifically, they convert cardioprotective high-density lipoprotein (HDL) into a dysfunctional, pro-atherogenic phenotype, further exacerbating the atherothrombotic progression. Furthermore, NET-bound neutrophil elastase (NE) specifically degrades tissue factor pathway inhibitor (TFPI), neutralizing endogenous anticoagulant defenses and rendering the thrombus highly resistant to fibrinolysis (74, 75).

### 5.3 Immunomodulatory factors and the bidirectional feedback loop

High-mobility group box 1 (HMGB1), a prominent NET-associated damage-associated molecular pattern (DAMP), is recognized and endocytosed by macrophages via the RAGE and TLR4 receptors (76). Once within the lysosomal compartment, HMGB1 triggers the activation of the caspase-1 axis to induce macrophage pyroptosis, thereby magnifying the inflammatory signaling cascade (76, 77).

This bidirectional crosstalk between NETosis and pyroptosis has been validated across various pathological contexts, notably sepsis and AS. Specifically, NETosis potentiates pyroptosis via the AIM2 inflammasome and NE-mediated GSDMD proteolytic processing; conversely, the inflammatory secretome and DNA liberated during pyroptosis reciprocally stimulate NET formation (13, 54). This self-perpetuating inflammatory cycle drives chronic tissue injury and serves as a fundamental pathological substrate for acute thrombotic events, such as acute coronary syndromes.

## 6 Therapeutic interventions targeting NETs and interconnected pathways

Given the pivotal role of NETs in bridging autoimmune and cardiovascular pathologies, therapeutic strategies targeting their biogenesis, clearance, and downstream sequelae hold substantial clinical promise. Current therapeutic paradigms are categorized into three primary axes: upstream inhibition of NETosis, disruption of the NETosis–pyroptosis reciprocal loop, and the clearance or neutralization of mature NET structures (78–80).

Concerning the upstream inhibition of NETosis, pharmacological modulation of key enzymatic drivers and oxidative stress pathways has exhibited significant therapeutic potential. PAD4 is instrumental in mediating histone citrullination and the subsequent decondensation of chromatin. Genetic ablation of PAD4 or the administration of small-molecule inhibitors—notably Cl-amidine, GSK484, and GSK199—significantly mitigates NET formation and attenuates the atherosclerotic burden (78, 81). Similarly, the NE inhibitor sivelestat effectively suppresses NET liberation by impeding the nuclear translocation of NE (14). To counteract oxidative stress-driven suicidal NETosis, antioxidants such as N-acetylcysteine (NAC) or NADPH oxidase (NOX2) inhibitors are employed to modulate ROS levels (82). Furthermore, the microtubule inhibitor colchicine exerts pleiotropic effects by disrupting cytoskeletal dynamics and inhibiting NLRP3 inflammasome activation, thereby concurrently reducing NETosis and thrombotic risk (78, 83).

Given the self-perpetuating feedback loop between pyroptosis and NETosis, targeting the pyroptotic executioner GSDMD and its associated inflammasomes has emerged as a pivotal strategy to disrupt this pathogenic cycle. Disulfiram, an FDA-approved agent for the treatment of alcohol dependence, was recently identified as an inhibitor of GSDMD pore formation. In atherosclerotic models, disulfiram not only suppresses pyroptosis and NET liberation but also attenuates IL-1β secretion and enhances the autophagic clearance efficiency of macrophages (79). A novel targeted inhibitor, GIY-2, directly impairs the membrane-binding affinity of the GSDMD-N domain by targeting the Arg10 residue, thereby effectively suppressing macrophage pyroptosis and plaque progression (84). Additionally, compounds with a pyrazolo-oxazepine scaffold have been demonstrated to bind GSDMD, thereby inhibiting the execution of NETosis (53). At the signaling level, NLRP3 inflammasome inhibitors (e.g., MCC950) indirectly mitigate NETosis by preventing inflammasome assembly, while IL-1β antagonists (Anakinra) and IL-18-binding proteins disrupt the NETosis–pyroptosis axis by intercepting cytokine-mediated feedback loops.

Beyond pharmacological blockade of biogenic pathways, the direct clearance or neutralization of extracellular NET components represents a vital therapeutic paradigm. Recombinant human DNase I (rhDNase I), currently indicated for cystic fibrosis, is being investigated for its capacity to enzymatically dissolve the DNA scaffold of NETs within atherosclerotic lesions (78). To counteract the cytotoxicity of histones and cationic proteins, unfractionated and low-molecular-weight heparins (LMWHs) utilize their high negative charge density to sequester histones, thereby destabilizing the NET scaffold and neutralizing its inherent toxicity. Furthermore, specific peptides targeting histone H2A can selectively interfere with the interaction between NETs and platelets, thereby mitigating the progression of atherosclerotic lesions (78). Moreover, burgeoning advancements in nanomedicine and metabolic regulation offer novel avenues for precision therapeutic interventions. For instance, the employment of macrophage-homing nanoparticles to deliver GSDMD inhibitors or anti-inflammatory agents can substantially elevate local drug concentrations while minimizing systemic off-target effects (84). Within the pathological context of RA, pharmacological inhibition of the NF-κB signaling axis has proven effective in suppressing the pyroptotic transformation of FLSs (29).

## 7 Discussion

This review systematically synthesizes the current understanding of NET formation and pyroptosis as two pivotal innate immune responses. By elucidating their intricate interactions, we establish these pathways as a fundamental molecular nexus bridging systemic autoimmune diseases with AS. Our analysis demonstrates that NETs serve not only as primary initiators and amplifiers of autoimmune responses but also as indispensable components of a self-reinforcing “NET–pyroptosis axis.” This reciprocal positive feedback loop plays a decisive role in perpetuating chronic inflammation, tissue injury, and thrombogenesis (8, 57). Collectively, these insights provide a novel pathophysiological framework for understanding the significantly heightened cardiovascular risk in patients with systemic autoimmunity, suggesting potential avenues for cross-disciplinary therapeutic innovation.

### 7.1 The NET–pyroptosis axis: a self-amplifying inflammatory hub

Historically, NETosis and pyroptosis were perceived as discrete programmed cell death modalities with divergent roles in host defense and inflammation (85). However, the evidence synthesized in this review clearly delineates a profound bidirectional regulatory nexus and synergistic amplification between these two pathways.

On one hand, NETs function as potent DAMPs. Their DNA scaffolds and tethered proteins, such as HMGB1, are recognized by myeloid cells—notably macrophages—leading to the activation of AIM2 or NLRP3 inflammasomes and the subsequent induction of GSDMD-dependent classical pyroptosis (13, 54). Conversely, the executioner protein GSDMD facilitates NETosis by permeabilizing the nuclear and granular membranes, effectively providing an expedited kinetic pathway for chromatin expulsion. This process is further intensified when NE within the NETs reciprocally cleaves GSDMD to potentiate its pore-forming activity, thereby establishing a robust positive feedback loop (23, 86). The conceptualization of this NET–pyroptosis axis bridges two potent pro-inflammatory mechanisms, elucidating why inflammatory responses in chronic pathologies—such as autoimmunity and AS—become recalcitrant and prone to self-sustained amplification once initiated.

Crucially, this pathogenic interplay extends beyond the hematopoietic compartment. Evidence from RA demonstrates that NETs can induce pyroptotic-like cell death in non-immune stromal cells, specifically FLSs, via a caspase-3/GSDME-mediated axis (29). This finding reveals a novel mechanism of NET-induced tissue destruction and suggests that the NET–pyroptosis axis operates across a diverse cellular and tissue spectrum. The specific manifestation of this axis—whether GSDMD- or GSDME-dominant—is likely dictated by the local inflammatory milieu and the specific cell lineages involved.

### 7.2 Disease-specific manifestations and the common pathological core

While the NET–pyroptosis axis represents a conserved pathological foundation across systemic autoimmune diseases and AS, its upstream triggers and downstream sequelae manifest with distinct disease-specific nuances. In SLE, the pathogenesis is fundamentally driven by persistent type I interferon (IFN-I) signaling and a loss of B-cell tolerance. Defective NET degradation facilitates the sustained exposure of endogenous antigens, which primes pDCs and B cells, thereby perpetuating a self-amplifying “NET–IFN-I–autoantibody” loop (18, 37). Conversely, the pivotal role of NETs in RA centers on the liberation of diverse citrullinated autoantigens, which directly triggers the production of anti-citrullinated protein antibodies (ACPAs). Furthermore, NET-induced pyroptosis of FLSs actively exacerbates synovial invasion and osteoarticular destruction (29, 87). In AS, metabolic DAMPs—notably cholesterol crystals and oxLDL—serve as the primary provocateurs of the NET–pyroptosis axis. The downstream pathological consequences are characterized by the sustained amplification of intra-plaque inflammation, endothelial dysfunction, and thrombogenesis (58).

This “common core, distinct triggers” paradigm offers significant clinical insights. The existence of a common pathological core suggests that pharmacological agents targeting the NET–pyroptosis axis may possess broad-spectrum therapeutic potential, enabling a “unified treatment” strategy across a range of clinical conditions. Concurrently, the diversity in initiating factors implies that targeting upstream, disease-specific triggers may facilitate more precise and individualized therapeutic interventions. This dual-pronged strategy could maximize therapeutic efficacy while addressing the specific requirements of distinct patient cohorts.

### 7.3 Therapeutic prospects, challenges, and future directions

Pharmacological targeting of the NET–pyroptosis axis represents a transformative frontier in the management of both systemic autoimmune disorders and AS. Current therapeutic interventions focus on a tripartite strategy: attenuating upstream NETosis through PAD4 or NE inhibitors, disrupting the core positive feedback loop via NLRP3 or GSDMD antagonists, and facilitating the enzymatic degradation of mature NET scaffolds using DNase I. Despite encouraging preclinical outcomes, several hurdles impede successful clinical translation (88, 89). Primary among these challenges is the imperative to preserve host defense mechanisms (90). Given that NETosis and pyroptosis are essential physiological processes for pathogen clearance, their systemic, chronic inhibition may compromise antimicrobial immunity and heighten the risk of opportunistic infections. Future interventions must strike a precise balance between mitigating pathological inflammation and maintaining physiological immune surveillance. The development of site-specific agents or stimuli-responsive drugs that target pathological niches will be pivotal in overcoming these safety concerns.

Moreover, the inherent robustness and redundancy of inflammatory networks pose a significant challenge. Monotherapy targeting a single molecular node may be insufficient to arrest the inflammatory cascade due to the activation of compensatory signaling pathways. For instance, pharmacological blockade of the NLRP3 inflammasome may be circumvented by the AIM2 sensor, which remains sensitive to NET-associated DNA. Consequently, synergistic combination therapies or pleiotropic agents targeting multiple nodal points within the axis may offer superior clinical efficacy. Future research efforts should prioritize the following three domains. First, the intricate regulatory landscape of the NET–pyroptosis axis must be delineated across diverse tissue microenvironments and neutrophil subpopulations, such as LDGs (91). Second, circulating biomarkers of NETosis, notably MPO-DNA complexes and Cit-H3, require validation in large-scale prospective cohorts to solidify their utility for risk stratification and therapeutic monitoring. Third, the integration of advanced drug delivery systems, such as nanomedicine, is essential for the site-specific delivery of inhibitors to atherosclerotic plaques or arthritic joints, thereby enhancing the local therapeutic index while minimizing systemic toxicity (92).

## 8 Conclusion

In conclusion, the NET–pyroptosis axis represents a fundamental pathological driver of chronic inflammation in both systemic autoimmune diseases and AS. The identification of this molecular nexus has profoundly reshaped our understanding of the pathogenic interplay between these conditions, unveiling unprecedented therapeutic opportunities. While significant clinical hurdles persist, the continued elucidation of the molecular mechanisms underlying this inflammatory axis—complemented by the development of innovative pharmacological strategies—provides a robust rationale for optimism. Targeting the reciprocal interaction between NETosis and pyroptosis is poised to yield transformative breakthroughs in clinical management. These advancements will be instrumental in improving the long-term prognosis of patients with autoimmune disorders and, most importantly, in mitigating the disproportionately high risk of cardiovascular complications within these populations.
